# Subchondral Bone Microarchitectural and Mineral Properties and Expression of Key Degradative Proteinases by Chondrocytes in Human Hip Osteoarthritis

**DOI:** 10.3390/biomedicines9111593

**Published:** 2021-11-01

**Authors:** Yunfei Li, Yulia Liem, Zaitunnatakhin Zamli, Niall Sullivan, Enrico Dall’Ara, Haroon Ahmed, Grace Matilda Sellers, Ashley Blom, Mohammed Sharif

**Affiliations:** 1 Musculoskeletal Research Unit, Translational Health Sciences, Bristol Medical School, University of Bristol, Bristol BS10 5NB, UK; yunfei.li@bristol.ac.uk (Y.L.); yulia.liem@bristol.ac.uk (Y.L.); Ashley.Blom@bristol.ac.uk (A.B.); 2 Department of Biomedical Science, Kulliyyah of Allied Health Sciences, International Islamic University Malaysia, Kuantan 25200, Pahang, Malaysia; zaitun@iium.edu.my; 3 Department of Trauma and Orthopaedics, Bristol Royal Infirmary, University Hospitals Bristol and NHS Foundation Trust, Bristol BS2 8HW, UK; niall.sullivan@uhbw.nhs.uk; 4 Department of Oncology and Metabolism and Insigneo Institute for In Silico Medicine, University of Sheffield, Sheffield S1 3JD, UK; e.dallara@sheffield.ac.uk; 5 Bristol Medical School, University of Bristol, Bristol BS8 1UD, UK; qb18128@bristol.ac.uk (H.A.); gs17013@bristol.ac.uk (G.M.S.); 6 National Institute for Health Research Bristol Biomedical Research Centre, University Hospitals Bristol and NHS Foundation Trust, University of Bristol, Bristol BS8 2BN, UK

**Keywords:** cartilage degradation, MMP13, ADAMTS4, subchondral bone remodelling, subchondral bone microarchitecture, tissue mineral density

## Abstract

Background: The purpose of this study was to investigate the relationship between the expression of key degradative enzymes by chondrocytes and the microarchitectural and mineral properties of subchondral bone across different stages of cartilage degradation in human hip osteoarthritis (OA). Methods: Osteochondral samples at different stages of cartilage degradation were collected from 16 femoral heads with OA. Osteochondral samples with normal cartilage were collected from seven femoral heads with osteoporosis. Microcomputed tomography was used for the investigation of subchondral bone microarchitecture and mineral densities. Immunohistochemistry was used to study the expression and distribution of MMP13 and ADAMTS4 in cartilage. Results: The microarchitecture and mineral properties of the subchondral plate and trabecular bone in OA varied with the severity of the degradation of the overlying cartilage. Chondrocytes expressing MMP13 and ADAMTS4 are mainly located in the upper zone(s) of cartilage regardless of the histopathological grades. The zonal expression of these enzymes in OA (i.e., the percentage of positive cells in the superficial, middle, and deep zones), rather than their overall expression (the percentage of positive cells in the full thickness of the cartilage), exhibited significant variation in relation to the severity of cartilage degradation. The associations between the subchondral bone properties and zonal and overall expression of these enzymes in the cartilage were generally weak or nonsignificant. Conclusions: Phenotypic changes in chondrocytes and remodelling of subchondral bone proceed at different rates throughout the process of cartilage degradation. Biological influences are more important for cartilage degradation at early stages, while biomechanical damage to the compromised tissue may outrun the phenotypic change of chondrocytes and is critical in the advanced stages.

## 1. Introduction

Osteoarthritis (OA) is one of the most common musculoskeletal conditions in the ageing population. It affects all components of a joint, including the cartilage, subchondral bone, and synovium [1]. OA causes joint pain and dysfunction, and is among the leading causes of disability [2]. There are no disease-modifying reagents for OA and most of the current treatments for OA are symptom-relieving.

The pathological mechanism(s) of OA is still unclear, but the interactions between cartilage and subchondral bone may play a critical role. Subchondral bone provides mechanical support for cartilage, absorbing and distributing mechanical forces [3]. Meanwhile, articular cartilage lubricates joint movements and prevents concentrated focal stress on subchondral bone [4]. Changes in the biomechanical properties of either of these two tissue types will inevitably affect the transmission of load to the other, which can be perceived by chondrocytes and bone cells and transmitted into intracellular signals, disrupting normal cell and matrix metabolism [5,6]. Chondrocytes and bone cells in OA may also exert regulatory effects on each other through biochemical factors (e.g., cytokines and growth factors), which can travel across the osteochondral junction via increased vascular channels and microcracks [5,7].

The microarchitecture and mineralization of bone are not only the determinants of its mechanical properties, but also reflect the remodelling activities of bone cells. Numerous studies on animal OA models have shown that cartilage degradation is accompanied by changes in the microarchitecture and/or mineralization of subchondral bone (reviewed in [8,9]). Several previous studies on human knees and hips have shown that the microarchitectural and mineral parameters of subchondral bone are closely associated with the local severity of cartilage degradation in OA [10,11,12,13,14,15,16]. Cartilage degradation, represented by the loss of matrix macromolecules, especially proteoglycans and type II collagen, is attributable to the proteolytic activities of aggrecanases and collagenases [17]. However, it is not yet clear to what extent these enzymes contribute to different stages of cartilage degradation in OA [18,19] and how their expression by chondrocytes and distribution in cartilage may be related to the microarchitectural and mineral properties of subchondral bone.

To our knowledge, few studies on animal models have examined subchondral bone properties and the expression of degradative enzymes in cartilage simultaneously [20,21,22,23], and to date there have been no studies in which the expression of degradative enzymes by chondrocytes was directly related to the microarchitecture and mineralization of subchondral bone in either human samples or animal models. Filling this knowledge gap may provide novel insights into the mechanisms and relationship between cartilage destruction and subchondral bone remodelling in OA; this is the aim of the current study. We first carried out a detailed investigation of the expression of degradative enzymes by chondrocytes and their in situ distribution at various stages of cartilage degradation in human hip OA, and then examined how these features of cartilage are associated with the properties of subchondral bone. We selected matrix metalloproteinase-13 (MMP13) and a disintegrin and metalloproteinase with thrombospondin motifs-4 (ADAMTS4) as representatives of collagenases and aggrecanases, respectively [17], and included samples from patients with osteoporosis (OP) for comparison.

## 2. Materials and Methods

### 2.1. Patient Selection

Sixteen patients with hip OA (six females and 10 males, mean age 68.1 ± 8.0 years) and seven patients with low-energy fracture of the hip due to OP (three females and four males, age 69.7 ± 5.9 years) who underwent hip arthroplasty surgeries were recruited at Southmead Hospital, Bristol, UK. Diagnosis of OA and OP was based on established guidelines [24,25]. Femoral heads excised during the surgeries were collected upon obtaining written consent from patients, which was approved by the Health Research Authority, UK (17/WS/0217, 2 November 2017). For the OA group, patients with rheumatoid arthritis, avascular necrosis, and known history of hip infection or trauma were excluded. For the OP group, patients who were on medications affecting bone metabolism or had secondary OP due to prolonged use of corticosteroids were excluded. Inclusion of femoral specimens also depended on a macroscopic evaluation of the articular surface, as described below.

### 2.2. Macroscopic Evaluation and Osteochondral Plug Extraction

**Figure 1:**
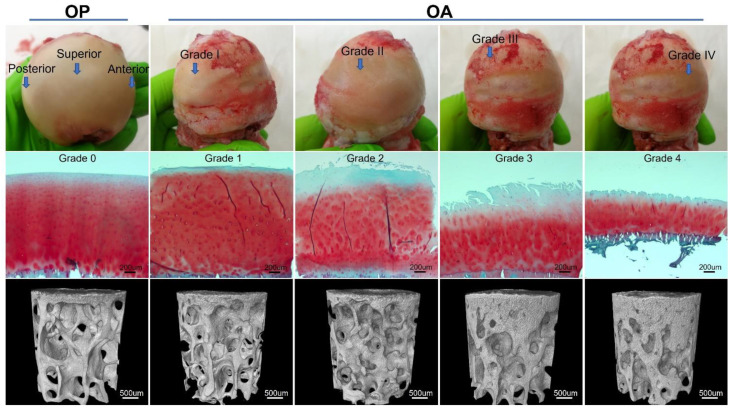
Macroscopic and microscopic evaluation of cartilage degeneration, and cylindrical region of interest for microCT analysis. Femoral specimens from patients with OP and OA are shown in the first panel. Images of the OA specimen show the same sample viewed from different angles. Arrows point to anatomical sites in OP specimen and regions with different macroscopic grades in OA specimen from which two immediately adjacent osteochondral plugs were extracted. Microscopic grading of the undecalcified paraffin embedded osteochondral samples using tissue sections stained with safranin O and fast green are shown in the second panel. The three-dimensional cylindrical primary region of interest of subchondral bone for analysis of microarchitecture and mineral properties are shown in the bottom panel.

The articular surface of OA femoral heads was regionally divided according to the macroscopic grading system of cartilage degradation reported in a recent publication [10]. Two immediately adjacent osteochondral plugs (50 pairs) with 4 mm diameter and ~10 mm height were collected from each graded region depending on availability using a steel hollow punch (Figure 1). The included regions ranged from Grade I (normal/comparatively normal) to Grade IV (severe cartilage erosion). Grade V, in which cartilage was completely depleted, was not included. For the OP group, femoral heads were included only when the articular surface fell into the Grade I category, then two immediately adjacent osteochondral plugs were extracted from the superior, anterior, and posterior sites (21 pairs).

### 2.3. MicroCT Scanning

One set of the paired plugs was stored at −80 °C, until they were transported to the collaborating lab (University of Sheffield) for microCT scanning. These plugs were a subset of the samples used in a previous study and were scanned by Skyscan 1172 (Kontich, Belgium), as previously described [10]. Briefly, images with an isotropic voxel size of 4.87 µm were acquired using the following scanning parameters: 50 KeV voltage, 179 µA intensity, 1180 ms integration time, and 180° rotation. Beam-hardening artifacts were reduced using a 0.5-mm aluminium filter. Three-dimensional reconstruction of datasets was carried out by NRecon software (1.6.9.4, Skyscan). Two calcium hydroxyapatite phantoms with known mineral densities (0.25 and 0.75 g/cm^3^) were scanned and reconstructed under the same conditions.

### 2.4. MicroCT Image Analysis

The CT Analyzer software (CTAn, 1.17.7.2, Skyscan) was used for image analysis. Segmentation between bone and soft tissue/marrow was based on a global binarization threshold. A preliminary cylindrical region of interest (ROI) with 3.0 mm diameter and 4.0 mm depth was chosen for the subchondral bone of each sample (Figure 1), which was then separated into ROIs of subchondral plate and trabecular bone using a semi-automated protocol, as described previously [10].

The following microarchitectural parameters of subchondral trabecular bone were calculated by the CTAn according to established guidelines [26]: bone volume fraction (BV/TV), specific bone surface (BS/BV), trabecular thickness (Tb.Th), trabecular number (Tb.N), structural model index (SMI), and connectivity density (Conn.Dn). The thickness of the subchondral plate (Pl.Th) was also calculated by applying the sphere-fitting method [26] to the contour of the ROI of the subchondral plate.

Hydroxyapatite phantoms were used to define a linear densitometric calibration equation to convert the X-ray attenuation coefficient values into tissue mineral density (TMD). TMD, which reflects the mean degree of bone matrix mineralization, was measured as mineral content over the volume of bone tissue, excluding pores and marrow space [12,26]. TMD was calculated for the subchondral plate (Pl.TMD) and trabecular bone (Tb.TMD).

### 2.5. Histological Processing and Microscopic Grading

The second set of paired plugs was fixed in formalin for 24 h immediately after extraction. The subchondral bone of these plugs was trimmed to about 1.0 mm in length without damaging the overlying cartilage. They were then processed for paraffin embedding without decalcification. Osteochondral tissue sections that were 7 µm thick were produced.

Duplicate sections randomly selected from each plug were subjected to safranin O-fast green staining [27]. Microscopic grading of cartilage degradation was carried out using a modified version [10] of the OARSI grading system [28] by two observers (Y. Li and Y. Liem) in a blinded manner. Scoring was repeated after eight weeks and both inter- and intra-observer intraclass correlation coefficients (ICCs) were above 0.94, demonstrating excellent reliability [29,30]. Grades 5 and 6 were not present as samples with complete cartilage loss were excluded from the study. Microscopic grades of these samples were compared with those of the paired plugs scanned by microCT, as reported in [10], to make sure the microscopic grade was the same between the paired adjacent plugs collected from each macroscopically graded region.

### 2.6. Immunohistochemical Detection of MMP13 and ADAMTS4

Paraffin-embedded tissue sections were used to detect MMP13 and ADAMTS4 expression by chondrocytes using immunohistochemistry (IHC). Reagents were purchased from Abcam (Cambridge, UK). Duplicated sections were deparaffinized and rehydrated. Then heat-induced antigen retrieval was carried out in a citrate buffer at 95 °C for 10 min. This was followed by blocking endogenous peroxidase activities with a 3% hydrogen peroxide solution. Next, 10% normal goat serum (ab7481) was applied to tissue sections for 1 h to prevent nonspecific antibody binding. Then polyclonal rabbit primary antibody (IgG) against MMP13 (1:300, ab39012) or ADAMTS4 (1:200, ab84792) was applied and incubated overnight at 4 °C. Additional sections were incubated with a blank buffer or with nonimmune rabbit IgG (ab37415) as negative control (Figure S1). The next day, sections were washed and treated with biotinylated goat anti-rabbit secondary antibody (ab64256) for 30 min. This was followed by treatment with streptavidin–horseradish peroxidase (HRP) conjugates (ab64269). Chromogenic detection was then carried out with diaminobenzidine (DAB) chromogen and a substrate (ab64238), which produced brown precipitates in chondrocytes expressing ADAMTS4 and MMP13. Finally, sections were counterstained with Harris haematoxylin.

### 2.7. Measurement of MMP13 and ADAMTS4 Expression

Slides were observed and photographed with a DM5500 microscope and digital camera (Leica Microsystems, Milton Keynes, UK). The number of chondrocytes positively and negatively stained with MMP13 or ADAMTS4 was counted manually at 100× magnification. A ROI was selected in the middle of sections with a width of 2 mm. The overall expression was expressed as a percentage of positively stained cells through the full thickness of cartilage in the ROI. The zonal expression was defined as the percentage of positive cells in the superficial zone (SZ), middle zone (MZ), and deep zone (DZ), respectively. Zones were differentiated according to characteristic chondrocyte morphology and alignment [4]. The availability of zones varied with the severity of cartilage degradation, i.e., SZ was lost and therefore not counted in microscopic Grade 3, and SZ and MZ were not counted in Grade 4. The counting was repeated after eight weeks on 50 randomly selected sections to evaluate intra-observer variability. The ICCs for the percentage of positively stained cells were 0.906, 0.975, 0.943, and 0.954 for the SZ, MZ, DZ, and full thickness of cartilage, respectively.

### 2.8. Statistical Analysis

Data are presented as the mean ± standard deviation (SD) unless otherwise indicated. The normal distribution of data was inspected using the Shapiro–Wilk test. An unpaired Student’s *t*-test and one-way ANOVA with a Bonferroni post hoc test were used for parametric data. Mann–Whitney U (M–W) and Kruskal–Wallis (K–W) tests with Dunn’s post hoc test were used for nonparametric data. Intragroup comparisons of proteinase expression were made between the SZ, MZ, and DZ for OP and each grade of OA. Intergroup comparisons of subchondral bone properties and proteinase expression were first made between different microscopic grades in the OA group to investigate variations related to severity of cartilage degradation. Tests were then repeated to incorporate the OP group to compare OP with different grades of OA. The associations between expression of proteinases and subchondral bone properties in OP and OA were determined using Pearson’s correlation for parametric data and Spearman’s correlation for nonparametric data. Statistical significance was indicated by a two-tailed *p*-value less than 0.05. GraphPad Prism (8.3.0, GraphPad Software, San Diego, CA, USA) and IBM SPSS (26.0, IBM Corp., Armonk, NY, USA) were used for statistical analysis and graphing.

## 3. Results

### 3.1. Microarchitecture and Mineral Properties of Subchondral Bone

**Figure 2:**
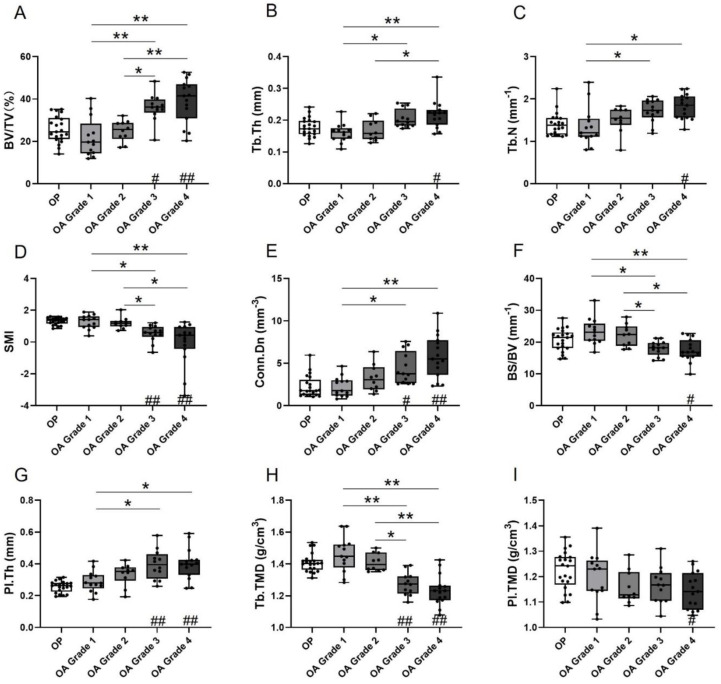
Microarchitectural and mineral properties of subchondral bone in OP and different stages of cartilage degradation in OA. (**A**) Volume fraction (BV/TV). (**B**) Trabecular thickness (Tb.Th). (**C**) Trabecular number (Tb.N). (**D**) Structural model index (SMI). (**E**) Connectivity density (Conn.Dn). (**F**) Specific bone surface (BS/BV). (**G**) Subchondral plate thickness (Pl.Th). (**H**) Tissue mineral density of trabecular bone (Tb.TMD). (**I**) Tissue mineral density of subchondral plate (Pl.TMD). The boxplots show the median, interquartile range, and individual values of the parameters measured. Results of intergroup comparisons between OA grades (statistical significance indicated by *) and between OP and OA grades (statistical significance indicated by #) are shown, where * *p* < 0.05; ** *p* < 0.001; the same for #.

Data for microarchitectural and mineral properties of subchondral bone in OA and OP groups are summarized in Table 1 and presented in Figure 2. For microarchitecture, within the OA group, the BV/TV, Tb.Th, Tb.N, and Conn.Dn of trabecular bone and thickness of subchondral plate (Pl.Th) exhibited a statistically significant increasing trend toward more severe degradation of the overlying cartilage (ANOVA *p* < 0.01 for BV/TV, Tb.Th, Tb.N, and Pl.Th; K–W test; *p* < 0.001 for Conn.Dn). The values of these parameters for OA Grade 1 and/or Grade 2 were significantly lower than those of Grades 3 and 4, as indicated by the post hoc analysis (Figure 2A–C,E,G). In contrast, SMI and BS/BV displayed a significant decreasing trend with the increasing severity of OA (*p* < 0.001, K–W test and ANOVA respectively), with Grades 1 and 2 showing significantly higher values than Grades 3 and 4 (Figure 2D,F). As for the mineral properties, the TMD of trabecular bone showed a decreasing trend with the increasing microscopic grade in OA (ANOVA, *p* < 0.001; Bonferroni test, *p* < 0.001 for OA Grade 1 and 2 vs. Grade 3 and 4) (Figure 2H). However, the differences in TMD of the subchondral plate between OA grades were not significant (Figure 2I). When compared between OP and different OA microscopic grades, the microarchitectural and mineral properties of subchondral bone in OP were similar to those in early stages of OA cartilage degradation (Grades 1 and 2), but significantly different from those in advanced stages of cartilage degradation (Grades 3 and/or Grade 4) (Figure 2).

### 3.2. Expression of MMP13 and ADAMTS4 by Chondrocytes

**Figure 3:**
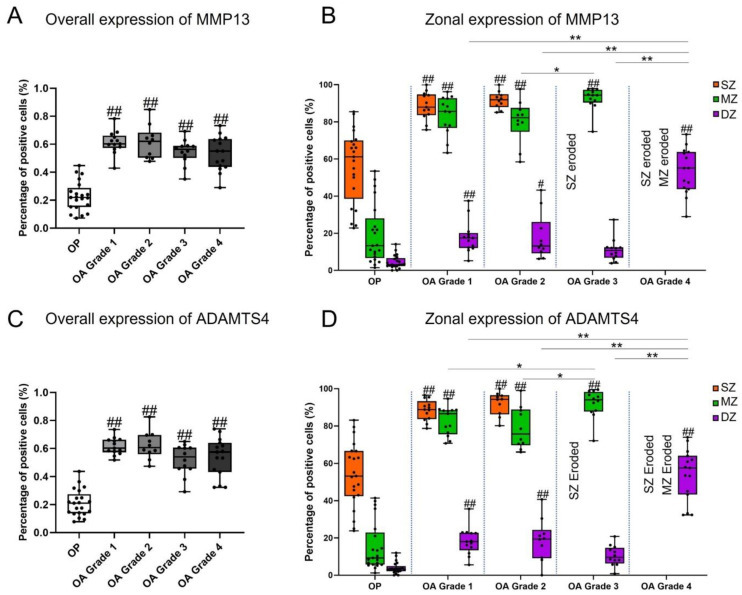
Overall and zonal expression of MMP13 and ADAMTS4 in OP and different stages of cartilage degradation in OA. The box plots show the median, the interquartile range (IQR), and individual values of percentage of chondrocytes stained positive for MMP13 (**A**,**B**) and ADAMTS4 (**C**,**D**). Results of intergroup comparisons between OA grades (statistical significance indicated by *) and between OP and OA grades (statistical significance indicated by #) are shown for overall expression (**A**,**C**) and zonal expression (**B**,**D**), respectively. * *p* < 0.05; ** *p* < 0.001; the same for #. SZ, superficial zone; MZ, middle zone; DZ, deep zone.

**Figure 4:**
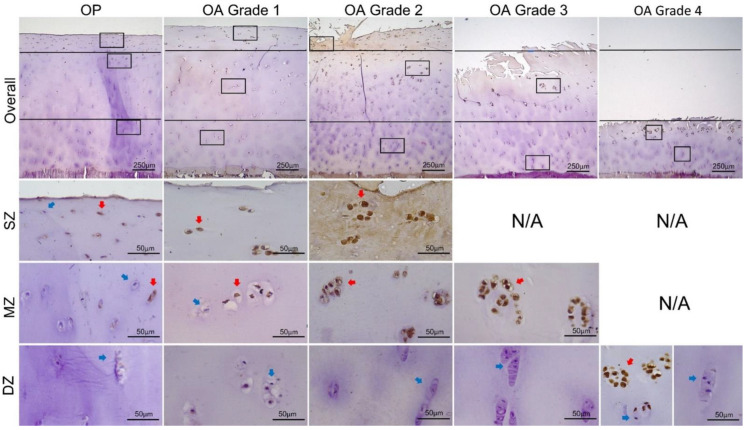
Representative images of IHC staining for MMP13 in OP and different stages of OA. The overall expression through the full thickness of cartilage is presented in the first panel at 40× magnification. Lines indicate separation of superficial (SZ), middle (MZ), and deep (DZ) zones and squares indicate higher-magnification (400×) areas in the subjacent panels. Blue and red arrows point to typical negatively and positively stained cells, respectively.

**Figure 5:**
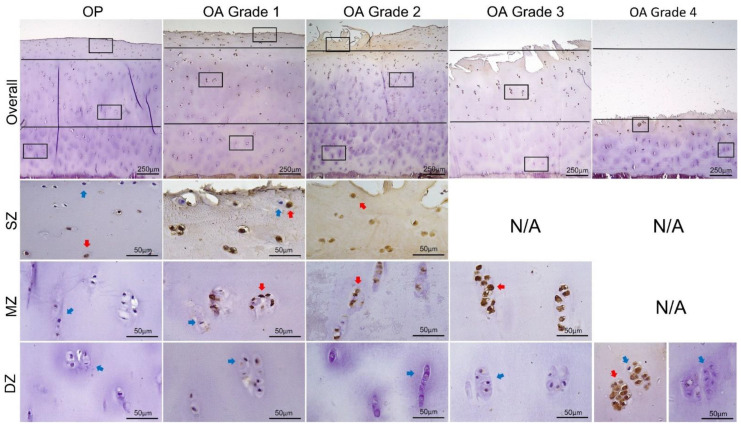
Representative images of IHC staining for ADAMTS4 in OP and different stages of OA. The overall expression through the full thickness of cartilage is presented in the first panel at 40× magnification. Lines indicate separation of superficial (SZ), middle (MZ), and deep (DZ) zones and squares indicate higher-magnification (400×) areas in the subjacent panels. Blue and red arrows point to typically negatively and positively stained cells, respectively.

Overall and zonal expressions of MMP13 and ADAMTS4, represented by percentage of positively stained chondrocytes in the full depth and in each zone of cartilage independently, are summarized in Table 2 and illustrated in Figure 3. Results of intragroup comparisons between SZ, MZ, and DZ are indicated in Table 2, and results of intergroup comparisons between different OA grades and between OP and OA are indicated in Figure 3. Representative images of staining are given in Figure 4 and Figure 5.

Within the OA group, there was no statistically significant difference in the overall expression of MMP13 and ADAMTS4 by chondrocytes between different microscopic grades of cartilage degradation (all around 60%; ANOVA, *p* > 0.05). The overall expression of the two enzymes in all OA grades was significantly higher compared with the OP group (Bonferroni test, *p* < 0.001) (Figure 3A,C).

In both OP and OA group, expression of MMP13 and ADAMTS4 was significantly higher in the upper zone(s) (Table 2; Figure 3B,D). In the OP group, the percentage of chondrocytes expressing MMP13 and ADAMTS4 in the SZ (56.39 ± 19.62% and 53.90 ± 17.34%, respectively) was significantly higher than that in the MZ (19.25 ± 16.11% and 15.17 ± 12.96%, respectively) (Dunn’s test, *p* < 0.001), and the MZ had significantly higher values compared to the DZ (4.45 ± 3.58% and 3.93 ± 3.11%, respectively) (Dunn’s test, *p* < 0.05). In OA Grades 1 and 2, expression of MMP13 and ADAMTS4 in the DZ (around 18%) was significantly lower compared to the MZ (around 80%) (Bonferroni test, *p* < 0.001), while there was not a significant difference between MZ and SZ (around 90%), except for ADAMTS4 in Grade 2, the expression of which was significantly lower in the MZ compared with SZ (Bonferroni test, *p* < 0.05). In OA Grade 3, the SZ was depleted and there were significantly more chondrocytes expressing MMP13 and ADAMTS4 in the MZ (92.52 ± 6.34% and 92.00 ± 7.76%, respectively) compared to DZ (11.07 ± 6.26% and 10.48 ± 5.46, respectively) (M–W test, *p* < 0.001). In OA Grade 4, SZ and MZ were not counted and 53.17 ± 12.37% and 53.86 ± 13.84% of chondrocytes expressed MMP13 and ADAMTS4 in the DZ, respectively.

Unlike the overall expression, the zonal expression of MMP13 and ADAMTS4 by chondrocytes exhibited significant variations in relation to the microscopic grades of cartilage degradation in OA (Figure 3B,D). The expression of MMP13 in the MZ was significantly higher in Grade 3 compared to Grade 2, and the expression of ADAMTS4 was significantly higher in Grade 3 compared to Grades 1 and 2 (Bonferroni test, *p* < 0.05). The percentage of chondrocytes expressing MMP13 and ADAMTS4 in the DZ in Grade 4 was significantly increased in contrast to those in Grades 1–3 (Bonferroni test, *p* < 0.001). MMP13- and ADAMTS4-expressing chondrocytes in the SZ and MZ of OP cartilage were significantly fewer compared to those in OA Grades 1, 2, and/or 3 (Bonferroni test, *p* < 0.001) (Figure 3B,D). Similarly, the expression of the two enzymes in the DZ was lower in OP compared with all grades of OA and was statistically significant for Grades 1, 2, and 4 (Bonferroni test, *p* < 0.001) (Figure 3B,D).

### 3.3. Correlations between Expression of MMP13 and ADAMTS4 and Subchondral Bone Properties

Results of correlations between the expression of ADAMTS4 and MMP13 by chondrocytes and the microarchitectural and mineral properties of subchondral bone are summarized in Table 3.

For the OP group, no statistically significant associations were found for either ADAMTS4 or MMP13. For the OA group, a few subchondral bone parameters were found to be significantly associated with either zonal or overall expression of the two enzymes, but the correlations were generally weak, with a *p*-value just below 0.05 and correlation coefficients around 0.3. Specifically, the overall expression of ADAMTS4 in OA cartilage was significantly and negatively associated with BV/TV and Tb.Th, and positively with the Tb.TMD (*p* = 0.049, 0.03, and 0.02; Pearson’s correlation coefficient = −0.28, −0.30, and 0.33, respectively). The expression of MMP13 in the DZ of cartilage was significantly associated with BV/TV, Tb.Th, and Tb.TMD (*p* = 0.049, 0.03, and 0.02; Spearman’s correlation coefficient = 0.28, 0.31, and −0.34, respectively). The overall expression of MMP13 in full-thickness OA cartilage was associated with Conn.Dn of trabecular bone (*p* = 0.01; Spearman’s correlation coefficient = −0.35).

## 4. Discussion

This is the first study in which the expression of the matrix-degrading proteinases by chondrocytes and the microarchitectural and mineral properties of subchondral bone across different stages of cartilage degradation have been studied concurrently in human OA samples and the associations between these parameters investigated. The data showed that chondrocytes expressing MMP13 and ADAMTS4 are mainly located in the upper zone(s) of cartilage, regardless of the histopathological grades. Zonal expression of these enzymes in OA, rather than their overall expression, exhibited a significant variation with the severity of cartilage degradation. It was also shown that the associations between subchondral bone properties and expression of these enzymes by chondrocytes were generally weak or not significant in OA and OP samples.

Subchondral bone microarchitecture and TMD results are generally in agreement with previous studies, showing that these parameters are closely associated with the conditions of the overlying cartilage in OA [10,11,12]. A thicker subchondral plate and volumetrically denser but hypomineralized trabecular bone were present in samples with advanced cartilage degradation (microscopic Grades 3 and 4). The comparisons of OA samples at various stages of cartilage degradation with OP samples support the notion that subchondral bone remodelling in OA is a dynamic process that favours resorption at early stages but is proformation, leading to sclerosis in late stages.

Moldovan et al. were among the first to report the localization of MMP13-expressing chondrocytes in human OA cartilage [31]. Consistent with the current study, they showed that the overall expression through the full thickness of cartilage was not significantly different between nonfibrillated and fibrillated regions. They also reported that the increased production of MMP13 in OA mainly came from chondrocytes in the DZ and lower MZ, and thus suggested that biological factors diffused from subchondral bone may be at least partially responsible for the phenotypic changes of chondrocytes in OA. However, this contrasts with the data presented here as well as in many other previous studies showing that MMP13 production was mainly located in the upper zone(s) irrespective of whether the sample had early or advanced cartilage degradation [32,33,34]. In addition, the steep drop in the percentage of positive cells from MZ to DZ in OA Grades 1–3 in this study corresponds to the sharp transition shown by Wu et al. [33] between areas displaying extensive staining and those with limited staining, suggesting a very slow progression of the phenotypic change in the OA chondrocyte population [33], which will be further discussed later.

Compared with MMP13, the localization of ADAMTS4 production in human OA cartilage is more consistent in the literature, i.e., predominantly in the upper zone(s) [34,35,36], as was shown by our study. This is in line with the universal observation that the loss of proteoglycans is most significant at and near the articular surface across various stages of OA, as demonstrated by the reduced cationic staining [28]. It is also worth noting that the zonal distribution of ADAMTS4 production was similar to that of MMP13 (Figure 3, Figure 4 and Figure 5), supporting the suggestion that these two enzymes are co-expressed by the same group of chondrocytes in OA [34], a group termed the “degradative chondrocytes” [34,36]. Such co-expression can be further verified either by staining the consecutive tissue sections, or by double antibody labelling. However, this is beyond the scope of the current study.

The findings that both ADAMTS4 and MMP13 are produced by chondrocytes located in the upper zone(s) are supportive of the concept that cellular and biochemical alterations in OA cartilage starts in the tissue closest to the articular surface [28,33]. With matrix degradation advancing toward subchondral bone and with the increasing severity of cartilage damage, it was expected that chondrocytes in the deeper layers of cartilage would become affected and exhibit the degradative phenotype [33,34,36] by starting to express proteinases that are not seen or are at lower levels in healthy adult cartilage [37,38]. However, the data presented here show that chondrocytes in the DZ that were not expressing MMP13 and ADAMTS4 in OA Grades 1 and 2 were still ‘clean’ of staining when matrix loss progressed into the MZ (Grade 3). More surprisingly, even when matrix degradation developed into the DZ (Grade 4) and there was just a thin layer of cartilage left, almost half of the surviving chondrocytes, located mainly in the lower DZ next to subchondral bone, were still negatively stained and displayed relatively normal morphology. In Grades 3 and 4, chondrocytes expressing MMP13 and ADAMTS4 increased in the MZ and DZ, respectively, but the positively stained cells were located almost exclusively in clusters near the surface and fissures (Figure 4 and Figure 5). These observations explain why the overall expression of the enzymes did not vary significantly between OA grades and may have important implications.

First, a theoretical model of cartilage degradation in OA can be suggested. Initially, the degradation of the extracellular matrix (ECM) is driven largely by the proteolysis of macromolecules, as a result of the phenotypic change of chondrocytes in the upper zones with increased production of cytokines, collagenase(s), and aggrecanase(s). Once the ECM is compromised, cartilage loses its unique elastic and frictionless properties and becomes more susceptible to mechanical destruction. The mechanical wear of the cartilage with inferior quality advances toward deeper layers quickly, while the shift of chondrocyte phenotype may be a much more gradual process [33]. At a certain stage of progression (likely between Grades 2 and 3), the directly damaging effects of mechanical forces may outrun those of biochemical disruption and reach the deeper layers of cartilage before subjacent chondrocytes develop a degradative phenotype. Then, at the late stage of cartilage degradation (Grade 4), erosion of the last portion of cartilage leading to the exposure of subchondral bone (Grade 5) may be largely or purely mechanical rather than biological. Several earlier studies have highlighted that cartilage degradation in OA is not simply a consequence of ‘wear and tear’ but a condition resulting from both biological and biomechanical reactions, with the former being more important [2,28,39,40,41,42]. The model presented here is consistent with these previous studies but additionally suggests that the relative contribution and importance of biological factors and mechanical disruptions to cartilage degradation and matrix loss may vary at different stages.

Second, it seems that biochemical factors (e.g., growth factors and cytokines) that induce a phenotypic change of chondrocytes are mainly produced by chondrocytes in the upper zones to act in an autocrine or paracrine manner, and/or from the synovium rather than from subchondral bone. If this was not the case, then chondrocytes in the DZ, which are closest to subchondral bone and are subjected to vascular invasion, should be affected at least at some point in the degradation process. Another possibility is that chondrocytes in the DZ may function as an intermediate of subchondral bone-derived signals rather than a direct effector that produces enzymes to degrade the ECM.

Subchondral bone is important for the maintenance of cartilage physiology in normal joints and contributes to cartilage pathology in disease conditions such as OA. It fulfils such roles by affecting the distribution of load in the overlying cartilage and through the cellular and biochemical crosstalk via the osteochondral junction. However, in this study, a statistically significant correlation between cartilage enzyme expression (overall and zonal) and subchondral bone microarchitectural and mineral properties was only observed for a few of the parameters investigated and the associations were rather weak, with *p*-values between 0.01 and 0.05 and correlation coefficients just around 0.3. This was unexpected since both the zonal expression of MMP13 and ADAMTS4 by chondrocytes and the microarchitecture and mineralization of subchondral bone exhibited significant variations in relation to the degree of cartilage damage (microscopic grading). One possible explanation is that the efficiency of adaptation in response to various mechanical or biological stimuli is markedly different between cartilage and bone [43,44,45]. Bone remodelling is a rapid process; the duration of a typical remodelling cycle takes 3–6 months from osteoclast activation to the completion of bone formation by osteoblasts [46]. On the contrary, the turnover of cartilage matrix components, especially aggrecan and type II collagen, takes decades in normal conditions, reflecting the limited capacity of chondrocytes to modify their surrounding matrix [47]. Although the metabolic activities of chondrocytes are elevated in OA, they are still relatively slow and the phenotype transition may take years [33,43]. Therefore, the pathological changes in subchondral bone and chondrocytes are likely to be at a different pace throughout OA, despite the fact that they both contribute to cartilage degradation. This is complementary to the cartilage degradation model suggested above: the increased mechanical stress borne by cartilage, as a result of the rapidly deteriorating subchondral bone properties, may outrun the biochemical degradation at some point and erodes cartilage quickly before chondrocytes in the deeper areas can respond.

A limitation of this study is that two immediately adjacent osteochondral samples from each of the regions graded by severity of cartilage degradation were used for microCT scanning and IHC detection. Although we ensured that the paired samples had the same macroscopic and microscopic grading, it would have been better if IHC were carried out on the same samples scanned by microCT. In addition, the scanned samples went through several freeze–thaw cycles as we did not have a microCT device on-site, which caused detrimental effects on the histological quality and antigenicity of tissue. Moreover, this is a cross-sectional study that used the severity of cartilage degradation (histopathological grading) as an indication of different stages of OA [38]. The associations between subchondral bone properties and expression of degradative enzymes by chondrocytes should also be investigated in a longitudinal manner—for example, in animal models.

## 5. Conclusions

In conclusion, the microarchitectural and mineral properties of subchondral bone and zonal expression, rather than overall expression, of MMP13 and ADAMTS4 by chondrocytes exhibited a significant variation in relation to the local severity of cartilage degradation in OA. However, the associations between subchondral bone properties and expression of these enzymes in cartilage were generally weak or not significant. Based on these findings, as well as the characteristic distribution of proteinase-expressing chondrocytes, a theoretical model of cartilage degradation in OA can be suggested: biochemical influences are more important to ECM degradation at the initiation and early stages, while the mechanical wear of the compromised tissue is faster than the phenotypic change of chondrocytes and is critical in advanced stages.

## Figures and Tables

**Table 1:** Microarchitecture and mineral densities of subchondral bone.

	OP	OA Grade 1	OA Grade 2	OA Grade 3	OA Grade 4	*p*-Value (OA Grades Only)	*p*-Value (OP and OA Grades)
BV/TV (%)	25.64 ± 6.24	21.79 ± 9.12	24.95 ± 5.00	35.94 ± 6.53	39.17 ± 10.34	<0.001	<0.001
Tb.Th (mm)	0.18 ± 0.03	0.16 ± 0.03	0.17 ± 0.03	0.20 ± 0.03	0.22 ± 0.04	<0.001	<0.001
Tb.N (mm^−1^)	1.40 ± 0.27	1.35 ± 0.46	1.51 ± 0.31	1.72 ± 0.27	1.82 ± 0.29	0.004	<0.001
SMI (–)	1.31 ± 0.22	1.26 ± 0.43	1.20 ± 0.36	0.54 ± 0.52	−0.07 ± 1.34	<0.001	<0.001
Conn.Dn (mm^−3^)	2.25 ± 1.28	2.23 ± 1.18	3.26 ± 1.58	4.42 ± 1.87	5.82 ± 2.54	<0.001	<0.001
BS/BV (mm^−1^)	20.64 ± 3.46	23.35 ± 4.17	22.14 ± 3.48	18.02 ± 2.34	17.42 ± 3.52	<0.001	<0.001
Pl.Th (mm)	0.26 ± 0.04	0.29 ± 0.07	0.33 ± 0.07	0.40 ± 0.09	0.40 ± 0.10	0.003	<0.001
Tb.TMD (g/cm^3^)	1.41 ± 0.06	1.46 ± 0.11	1.41 ± 0.06	1.27 ± 0.07	1.23 ± 0.10	<0.001	<0.001
Pl.TMD (g/cm^3^)	1.22 ± 0.07	1.20 ± 0.10	1.16 ± 0.07	1.17 ± 0.07	1.15 ± 0.07	0.35	0.03

Values are mean ± SD. Analysis of variance (one-way ANOVA or Kruskal–Wallis test (SMI and Conn.Dn)) were first run between OA grades to investigate variations in relation to the severity of cartilage degradation in OA. Then the tests were repeated to include the OP group to compare OP with different OA grades.

**Table 2:** Summary of overall and zonal expression of MMP13 and ADAMTS4.

	OP	OA Grade 1	OA Grade 2	OA Grade 3	OA Grade 4
MMP13					
Overall	22.76 ± 10.57	61.22 ± 8.29	61.53 ± 11.82	54.55 ± 8.99	53.17 ± 12.37
SZ	56.39 ± 19.62	88.64 ± 7.33	91.73 ± 4.64	/	/
MZ	19.25 ± 16.11 **	83.59 ± 10.35	80.22 ± 11.82	92.52 ± 6.34	/
DZ	4.45 ± 3.58 *	17.90 ± 8.67 **	17.87 ± 12.55 **	11.07 ± 6.26 **	53.17 ± 12.37
ADAMTS4					
Overall	21.12 ± 9.48	61.59 ± 6.06	62.37 ± 10.02	51.96 ± 10.97	53.86 ± 13.84
SZ	53.90 ± 17.34	88.48 ± 5.59	92.07 ± 6.26	/	/
MZ	15.17 ± 12.96 **	82.82 ± 7.68	78.97 ± 11.26 *	92.00 ± 7.76	/
DZ	3.93 ± 3.11 *	18.38 ± 7.48 **	18.71 ± 11.52 **	10.48 ± 5.46 **	53.86 ± 13.84

Data are the percentage (mean ± SD) of positively stained chondrocytes. *Intragroup comparisons by post hoc tests (Bonferroni’s or Dunn’s) following analysis of variance (one-way ANOVA or Kruskal–Wallis) for OP and OA Grades 1 and 2 (MZ vs. SZ and DZ vs. MZ), and by Mann–Whitney U test for OA Grade 3 (DZ vs. MZ). * *p* < 0.05, ** *p* < 0.001. SZ, superficial zone; MZ, middle zone; DZ, deep zone.

**Table 3:** Correlations between the expression of MMP13 and ADAMTS4 by chondrocytes and subchondral bone microarchitecture and mineral densities.

	OP				OA			
	SZ	MZ	DZ	Overall	SZ	MZ	DZ	Overall
**MMP13**								
BV/TV	0.53 (−0.14)	0.19 (−0.30)	0.88 (0.04)	0.40 (−0.19)	0.06 (0.40)	0.17 (0.24)	0.049 (0.28) *	0.12 (−0.22)
Tb.Th	0.86 (−0.04)	0.56 (−0.13)	0.76 (0.07)	0.94 (0.02)	0.78 (0.06)	0.55 (0.10)	0.03 (0.31) *	0.14 (−0.21)
Tb.N	0.73 (−0.08)	0.20 (−0.29)	0.92 (0.02)	0.69 (−0.09)	0.10 (0.21)	0.17 (0.24)	0.55 (0.09)	0.14 (−0.21)
SMI	0.14 (0.34)	0.61 (0.12)	0.11 (0.36)	0.28 (0.25)	0.12 (−0.33)	0.30 (−0.31)	0.09 (−0.24)	0.2 (−0.18)
Conn.Dn	0.84 (0.05)	0.27 (−0.25)	0.35 (−0.22)	0.67 (−0.10)	0.10 (0.35)	0.86 (0.03)	0.11 (0.23)	0.01 (−0.35) *
BS/BV	0.54 (0.14)	0.36 (0.21)	0.99 (−0.00)	0.71 (0.08)	0.54 (−0.14)	0.37 (−0.16)	0.06 (−0.27)	0.14 (0.21)
Pl.Th	0.95 (−0.01)	0.16 (−0.32)	0.27 (−0.25)	0.60 (−0.12)	0.93 (−0.02)	0.87 (0.03)	0.37 (0.13)	0.20 (−0.18)
Tb.TMD	0.59 (0.13)	0.21 (0.29)	0.45 (0.17)	0.34 (0.22)	0.06 (−0.40)	0.06 (−0.32)	0.02 (−0.34) *	0.12 (0.22)
Pl.TMD	0.40 (−0.19)	0.78 (−0.07)	0.52 (0.15)	0.94 (−0.02)	0.47 (0.16)	0.96 (0.01)	0.51 (−0.10)	0.67 (0.06)
**ADAMTS4**								
BV/TV	0.74 (−0.08)	0.09 (−0.38)	0.99 (0.003)	0.45 (−0.18)	0.24 (0.25)	0.20 (0.22)	0.12 (0.22)	0.049 (−0.28) *
Tb.Th	0.72 (−0.08)	0.98 (0.01)	0.09 (0.38)	0.64 (0.11)	0.35 (0.21)	0.06 (0.32)	0.10 (0.24)	0.03 (−0.30) *
Tb.N	0.98 (0.01)	0.30 (−0.28)	0.41 (−0.26)	0.23 (−0.28)	0.51 (0.14)	0.57 (0.10)	0.36 (0.13)	0.22 (−0.18)
SMI	0.30 (0.24)	0.22 (0.28)	0.13 (0.34)	0.41 (0.19)	0.54 (0.13)	0.16 (−0.24)	0.15 (−0.21)	0.08 (0.25)
Conn.Dn	0.91 (−0.02)	0.15 (−0.33)	0.50 (−0.16)	0.27 (−0.25)	0.06 (0.40)	0.52 (0.11)	0.19 (0.19)	0.15 (−0.21)
BS/BV	0.52 (0.15)	0.65 (0.11)	0.18 (−0.31)	0.93 (−0.02)	0.50 (−0.15)	0.05 (−0.33)	0.15 (−0.21)	0.06 (0.26)
Pl.Th	0.33 (0.22)	0.58 (−0.13)	0.73 (0.08)	0.63 (0.11)	0.28 (0.24)	0.17 (0.24)	0.18 (0.19)	0.67 (−0.06)
Tb.TMD	0.91 (0.03)	0.11 (0.36)	0.96 (0.01)	0.57 (0.13)	0.25 (−0.25)	0.21 (−0.22)	0.08 (−0.25)	0.02 (0.33) *
Pl.TMD	0.74 (−0.08)	0.78 (−0.06)	0.93 (−0.07)	0.84 (0.05)	0.31 (−0.22)	0.82 (−0.04)	0.05 (−0.28)	0.41 (−0.12)

Associations between expression of MMP13 and ADAMTS4 and subchondral bone properties were determined using Pearson’s correlation or Spearman’s correlation (underlined). Data are given as *p*-values (correlation coefficients). Statistically significant results are indicated by *.

## Data Availability

Data are contained within the article.

## Supplementary Materials

The following are available online at https://www.mdpi.com/article/10.3390/biomedicines9111593/s1, Figure S1: Specificity of immunohistochemistry staining.
