# Supplementary material for: Subchondral Bone Microarchitectural and Mineral Properties and Expression of Key Degradative Proteinases by Chondrocytes in Human Hip Osteoarthritis
Source: Biomedicines. 2021 Nov 1;9(11):1593. doi: 10.3390/biomedicines9111593 (PMC8615609; doi:10.3390/biomedicines9111593)
Supplement: Supplementary file 1 [file biomedicines-09-01593-s001.zip › biomedicines-1411467-supplementary.pdf]

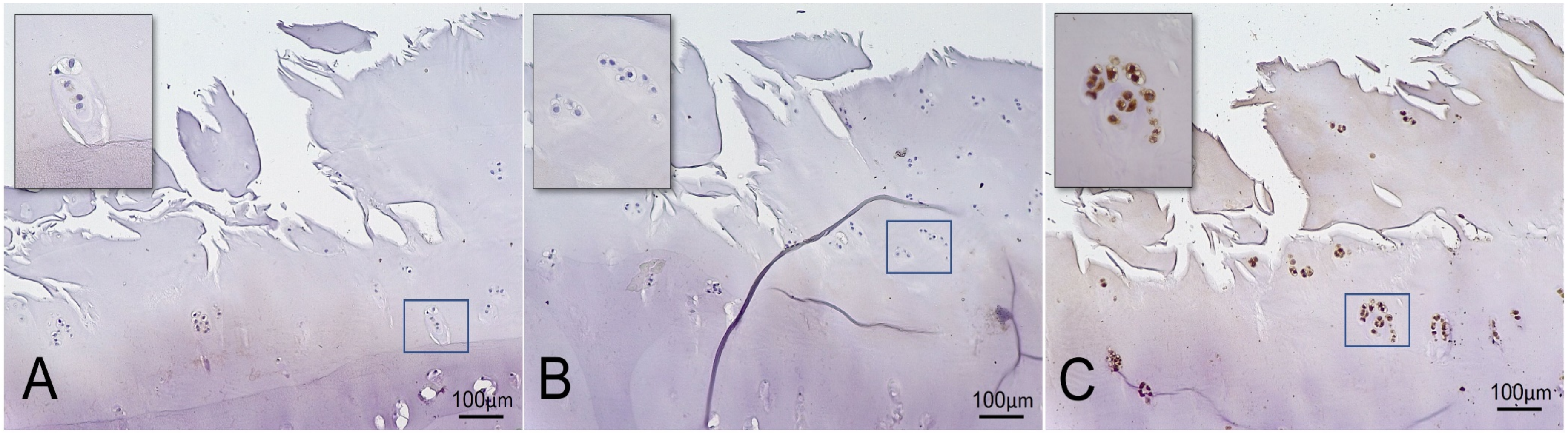

Figure S1. Specificity of immunohistochemistry staining. Tissue sections were incubated with blank diluent (**A**) and non-immune rabbit IgG (**B**) as negative antibody control. OA cartilage stained with anti-MMP13 primary antibody is shown in (**C**). Negligible background was observed in controls and specific staining can be seen in chondrocytes. Original magnification 100x. Squares indicate areas shown with 400x magnification.
